# The Evolving Use of Gold Nanoparticles as a Possible Reversal Agent for the Symptoms of Neurodegenerative Diseases: A Narrative Review

**DOI:** 10.7759/cureus.64846

**Published:** 2024-07-18

**Authors:** Alan D Kaye, Kelly R Sala, Drew Dethloff, Matthew Norton, Corey Moss, Michael J Plessala, Alyssa G Derouen, Yair Lopez Torres, Julian Kim, Sridhar Tirumala, Sahar Shekoohi, Giustino Varrassi

**Affiliations:** 1 Department of Anesthesiology, Louisiana State University Health Sciences Center, Shreveport, USA; 2 School of Medicine, Louisiana Health Sciences Center New Orleans School of Medicine, New Orleans, USA; 3 School of Medicine, Louisiana State University Health Sciences Center, Shreveport, USA; 4 Department of Pain Medicine, Paolo Procacci Foundation, Rome, ITA

**Keywords:** gold nanoparticles cancer, gold nanoparticles, gold flakes, cnm-au8, gold nanoparticles als, gold nanoparticles alzheimer’s, gold dust multiple sclerosis, gold dust parkinson’s disease

## Abstract

Neurodegenerative diseases are broadly hallmarked by impaired energy metabolism and toxic intracellular accumulations such as damaged organelles or reactive oxygen species (ROS). Gold nanoparticles readily cross the blood-brain barrier and increase nicotinamide adenine dinucleotide + hydrogen (NADH) oxidation to nicotinamide adenine dinucleotide (NAD+), which is vital for intracellular energy generation, cellular repair, and protection from ROS. Thus, the use of gold nanoparticles to treat and potentially reverse cellular injury seen in neurodegenerative disease has been an area of ongoing research. This systematic review explores current literature regarding the use of gold nanoparticle therapy in the treatment of neurodegenerative diseases such as Parkinson’s disease, Alzheimer’s disease, amyotrophic lateral sclerosis (ALS), and multiple sclerosis (MS). In vitro studies of CNM-Au8 (Clene Nanomedicine, Salt Lake City, UT) have been shown to reduce TDP-43 aggregates associated with ALS. These studies also exhibited the neuroprotective effects of CNM-Au8 in rat primary neurons exposed to amyloid-beta peptides, which are associated with Alzheimer’s disease. In animal models of MS, oral delivery of CNM-Au8 was demonstrated to produce robust and significant remyelination activity, oligodendrocyte maturation, and expression of myelin markers. In these same MS animal models, CNM-Au8 improved the motor function of cuprizone-treated mice in both open-field and kinematic gait studies. Recent phase II trials of CNM-Au8 in 13 patients with Parkinson’s disease and 11 patients with stable relapsing MS demonstrated a statistically significant increase in the NAD+/NADH ratio across two cohorts. As the current data repeatedly suggest, these gold nanoparticles are efficacious for the treatment and reversal of symptoms across these varying neurodegenerative pathologies. Further opportunities exist for increasing human trials and eventually incorporating this new technology into existing treatment regimens.

## Introduction and background

In 2016, neurological disorders were identified as the leading cause of disability and the second leading cause of death in the world. As the population ages, the number of those affected by non-communicable neurologic disorders, like Parkinson’s disease (PD), Alzheimer’s disease (AD), amyotrophic lateral sclerosis (ALS), and multiple sclerosis (MS) is expected to grow [1-4]. Despite the increasing burden of neurodegenerative disease, current therapies remain limited in number and efficacy.

PD is characterized by bradykinesia, rigidity, a “pill-rolling” resting tremor, and postural instability. Parkinson’s occurs as a result of the degeneration of dopamine-producing neurons in the substantia nigra. The resulting dysregulation of the nigrostriatal pathway causes increased inhibitory signals in the thalamus, followed by decreased excitatory input at the motor cortex, presenting as characteristic Parkinsonian symptoms. Current therapies aimed at treating PD include levodopa-carbidopa, dopamine agonists, and catechol-O-methyltransferase (COMT) inhibitors [5]. Although current treatments provide some symptomatic relief, they have not been shown to slow disease progression. These current treatments may also cause undesirable adverse effects such as nausea, vomiting, orthostatic hypotension, hallucinations, psychosis, fluctuations in motor function, and life-threatening cardiac arrhythmias [6]. They may also only offer symptom relief for a limited period. Levodopa-carbidopa is an example, as it generally only provides significant symptom improvement for the first five years of treatment.

AD is a neurodegenerative disease with insidious onset and inevitable progression, whose symptoms are dependent upon the stage of the disease. AD initially presents with episodic short-term memory loss, followed by impaired judgment and executive functioning. The middle stages of disease progression are associated with problems with multi-tasking and abstract thinking. Symptoms continue to become more severe, including psychosis, wandering, difficulty performing learned motor tasks, primitive reflexes, incontinence, and complete dependence on caregivers. AD occurs due to an accumulation of amyloid-beta (Aβ) plaque deposition in meningeal and cerebral vessels and gray matter, as well as the formation of neurofibrillary tangles within neuronal axons beginning in the hippocampus and progressing throughout the cortex. Significant neuronal loss in the nucleus basalis of Meynert, leading to low acetylcholine (ACh) levels, has also been observed. AD can have a strong autosomal dominant genetic component related to mutations in amyloid precursor protein (AAP), presenilin 1, and presenilin with near-complete penetrance. AD treatment options are limited to symptomatic treatment with cholinesterase inhibitors (e.g., Donepezil) to increase ACh concentration and the partial N-methyl-D-Aspartate (NMDA) antagonist, memantine. AD represents an especially large portion of the burden of neurodegenerative disease. The global prevalence is estimated to be nearly 24 million and is predicted to increase by 400% by 2050 [7].

ALS is a progressive nervous system disease characterized by upper and lower motor neuron symptoms that eventually result in paralysis and death. The exact cause of ALS is unclear, but multiple mechanisms have been proposed. Possible pathways resulting in cell death and disease are oxidative damage, mitochondrial dysfunction, caspase-mediated cell death (apoptosis), defects in axonal transport, abnormal growth factor expression, glial cell pathology, glutamate excitotoxicity, and aggregation of abnormal proteins [8]. These disease processes result in the degeneration of axons in the anterior and lateral columns of the spinal cord and the loss of motor neurons in the anterior horn of the spinal cord and Betz cells in the motor cortex. ALS treatment is mainly symptomatic, but some drugs aim to slow the disease process. Riluzole is the only drug that has been proven to improve survival. This is believed to be via reduced glutamate-induced cytotoxicity. Edaravone is a free radical scavenger that is also beneficial in slowing the decline among early-stage ALS patients. Even with these interventions, the median survival is three to five years, with only 30% of patients surviving five years [9].

MS is an autoimmune disease that affects the central nervous system, resulting in chronic inflammation, demyelination, gliosis, and neuronal loss. MS clinical presentation may vary from stable and chronic to rapid and debilitating. MS pathophysiology is associated with two major processes. The first is focal inflammation, which causes plaque formation and damage to small veins and the blood-brain barrier. The second process is neurodegenerative damage to the CNS neurons, axons, and synapses. These injuries result in the cytotoxic release of free radicals and demyelination of axons. Related to the multifocal nature of MS, it can present a broad range of symptoms. These typically include vision loss or double vision (due to optic neuritis), vertigo, gait imbalance, dysarthria, dysphagia, weakness, tremor, spasticity, fatigue, loss of sensation, paresthesia, incontinence, diarrhea, reflux, constipation, urinary urgency or retention, memory impairment, difficulty concentrating depression, and anxiety [10]. Disease-modifying therapies are the primary treatment for MS cases with the relapsing-remitting form of the disease. Other forms of MS are primarily neurodegenerative processes, so disease-modifying treatment options are less effective and serve little benefit in most cases [11].

There is an increasing interest in using gold nanoparticles (NPs or AuNPs) as a possible reversal agent for treating these neurodegenerative diseases. Many neurodegenerative diseases have been shown to have impaired energy metabolism and reactive oxygen species accumulation. In neurodegenerative diseases of aging, glucose metabolism deteriorates, resulting in vastly impaired mitochondrial functions, the inability to clear damaged organelles, and toxic intracellular accumulations [12].

A recently developed gold NP that readily crosses the blood-brain barrier may offer a solution to reducing or reversing disease-mediated neuron metabolic damage. CNM-Au8 (Clene Nanomedicine, Salt Lake City, UT) is a clean-surfaced, faceted gold nanocrystal (AuNC) that does not need the addition of organic capping or stabilization, which reduces the risk of adverse effects due to organic agent deposition. CNM-Au8 was shown to have significantly increased catalytic activity in the oxidation of NADH to NAD+, a vital component in intracellular energy generation, cellular repair, and protection from ROS [2].

Novel gold NP therapies offer a promising therapeutic approach to the treatment of neurodegenerative diseases, but their efficacy in large-scale human trials is yet to be seen. The focus of this review is to evaluate the effectiveness and plausibility of gold NPs in the treatment of neurodegenerative diseases, namely, PD, AD, ALS, and MS.

## Review

Methods

This was a narrative review. The sources for this review include PubMed, Google Scholar, Medline, and ScienceDirect using the following keywords: gold dust PD, gold dust MS, GNPs AD, GNPs ALS, CNM-Au8, gold flakes, GNPs, and GNPs cancer.

Experimental studies

One experimental study tested the ability of gold NPs to inhibit the aggregation of Aβ protein, a pathological hallmark of AD [13]. This study compared the effect of NPs to citrate control on the extent of Aβ protein aggregation expressed in human neuroblastoma cells. The NPs used in this experiment varied in surface chemistry, charge, and diameter so that the effect of these factors on the inhibitory potential of NPs could be investigated. This study concluded that NPs had a statistically significant inhibitory effect on Aβ aggregation (p < 0.001) and, more specifically, the smaller, anionic NPs were superior inhibitors to their larger counterparts.

One cohort study evaluated the efficacy of gold NPs in human subjects with neurodegenerative disease. It examined the benefits of using gold NPs as a treatment for human subjects with PD and MS [3]. The NAD+/NADH ratio, a measure of energetic capacity in the brain, was measured before and after 12+ weeks of treatment with CNM-Au8. CNM-Au8 is a drug with catalytic activity targeted at intracellular NADH conversion to NAD+. They started with two disease cohorts, 11 participants with stable relapsing MS and 13 with idiopathic PD, and conducted a cohort study. Phosphorus magnetic resonance spectroscopy (P-MRS) scans were performed at the initiation of the study and again at week 12 visits. Each participant received CNM-Au8 in a volume of 120 mL, dispensed in two 60 mL containers, and was instructed to drink both 60 mL bottles each morning on an empty stomach. Each disease cohort concordantly demonstrated increases in the NAD+/NADH ratio (mean increase of 10.4%). However, neither cohort reached statistical significance individually due to the small sample sizes (p = 0.11 and p = 0.14, PD and MS cohorts, respectively). The combined data from the two groups did reach statistical significance (SD = 1.3; p = 0.037, paired t-test). CNM-Au8 was well-tolerated in either cohort. Symptomatic improvement was not expected during this study due to its brevity and was not observed in either cohort.

Another finding from this study was that CNM-Au8 appears useful in achieving homeostatic equilibrium for both adenosine triphosphate (ATP) and the phosphorylation potential index, a ratio of high-energy to low-energy phosphates. Participants with lower baseline levels exhibited an increase in this metabolite energy index by week 12. Participants with higher baseline levels demonstrated a rebalancing effect, with levels equilibrating to the baseline population mean.

Another human study investigated the effects of CNM-Au8 on ALS patients with a 36-week phase 2, randomized, double-masked, placebo-controlled trial [14]. Participants (N = 45) were randomized 1:1 to receive either CNM-Au8 or a matching placebo daily over 36 weeks in addition to the background standard of care, riluzole. Participants were instructed to consume 60 mL of investigational medicinal product each morning on an empty stomach, orally or by feeding tube; for those in the treatment group, this daily medicinal product contained 30 mg of CNM-Au8. The primary outcome was mean percent change in the summed motor unit number index (MUNIX), a sensitive neurophysiological biomarker of lower motor neuron function. Although the patients in the treatment group exhibited a slower decline in MUNIX score compared to the placebo group, these results failed to reach statistical significance (least-squares mean difference: 20.9%; 95% CI: 2.2%-44.0%; p = 0.074). A secondary outcome of the study was all-cause mortality at 12 months. This was evaluated through an open-label extension (OLE), in which all participants were offered the option of continuing or initiating CNM-Au8 treatment upon completing the 36-week trial. Twenty of 22 participants were initially randomized to CNM-Au8, and 16 of 19 were originally randomized to placebo and entered the OLE. Disease progression was monitored in those who participated. Approximately 60% reduction in all-cause mortality over 12 months was observed in participants initially randomized to CNM-Au8 treatment (seven deaths in participants originally randomized to CNM-Au8 and 15 deaths in participants initially randomized to placebo). This difference was statistically significant (HR = 0.408, 95% Wald CI: 0.166 to 1.001, log-rank p = 0.0429). Throughout the study, CNM-Au8 was well-tolerated with no observed safety signals.

Further experimental trials were performed exclusively in rodent animal models rather than human test subjects. Still, the data obtained from these experiments are promising for using gold NPs in treating human neurodegenerative diseases. One such study measured the ability of CNM-Au8 to stimulate remyelination in mice [2]. The mice were treated with cuprizone, a demyelinating agent, before treatment with CNM-Au8. Degrees of axon myelination were then measured at different intervals. CNM-Au8 treatment consistently leads to higher levels of myelinated axons than vehicle-treated controls. CNM-Au8 treatment of cuprizone-injured animals enhanced the performance of the mice in each of the open-field test assays, restoring the observed cuprizone deficits versus sham-treated animals. Improvement in kinematic movement was also observed in CNM-Au8-treated mice relative to the sham controls. Most individual behavioral metrics were not statistically significant in either movement category.

This study used additional in vitro experiments to study the energy metabolism pathways involved. Oligodendrocyte precursor cells (OPCs) in culture were treated with AuNCs, which elevated levels of NAD+, intracellular ATP, and extracellular lactate. Myelin-synthesis-related genes were also upregulated. All these factors collectively resulted in enhanced myelin generation.

In another animal study, mice received seven days of continuous 1-methyl-4-phenyl-1,2,3,6-tetrahydropyridine (MPTP) intraperitoneal injection to induce PD symptoms before undergoing a series of tests measuring locomotor activities and motor coordination [15]. The mice were separated into two cohorts. One cohort received a low dose (5 mg kg^-1^) of AuNC treatment, and the other received a high dose (20 mg kg^-1^) of AuNC treatment. Both cohorts underwent the same series of tests again. The results were then compared to both their results after MPTP and before AuNC, as well as a separate control group that received neither. Compared to the control group, the PD model mice had both significantly reduced locomotor activities and inferior motor coordination (p < 0.01). The AuNC treatment reversed the decrease of locomotor activity and motor coordination caused by MPTP in a dose-dependent manner. The improvements reached statistical significance (p < 0.05) in most of the tests in the high-dose AuNC group. In contrast, the improvements were less pronounced in the low-dose AuNC group, which typically did not reach statistical significance (p > 0.05).

This study used additional in vitro experiments to show that AuNCs effectively prevent α-synuclein aggregation and fibrillation. It also used immunohistochemical and Western blot (WB) analyses to demonstrate that AuNCs can significantly reverse dopaminergic (DA) neuron loss in the substantia nigra and striatum of sick mice.

A similar study used MPTP to induce PD symptoms in mice and then injected them intra-abdominally with GNPs (2 mg Au/kg) to test their therapeutic value [16]. The substantia nigra-striatum system density was significantly reduced following injection with MPTP but began to recover with GNP therapy. Improvements were also seen when analyzing Nissl body concentration and a gait test after treatment with GNPs. Different GNP composites (5 and 10 nm) were used in separate cohorts, and more improvement was observed in each category for the 10 nm cohort compared to the 5 nm cohort.

Another study used 100 μg intracerebroventricular okadaic acid (OA) injections to create an AD model in male Wistar rats [17]. Some were treated with 20-nm AuNP at a dose of 2.5 mg/kg every 48 hours for 21 days. The following groups were separated (n = 12/group): sham, AuNP, OA, and OA + AuNP. Several of the deleterious effects caused by OA were shown to be reversed by AuNP. These included increased tau phosphorylation in the cortex and hippocampus, spatial memory impairment, oxidative stress, modulation of ATP synthase activity, and reduction of antioxidant capacities.

Discussion

Research into the potential applications of GNPs in treating neurological disorders is an area of active investigation. NPs can be designed to carry drugs across the blood-brain barrier, which is a major challenge in treating demyelinating neurological disorders. By encapsulating therapeutic agents within GNPs, it may be possible to enhance their delivery to specific brain regions affected by these diseases. These GNPs can be engineered to deliver therapeutic agents specifically to affected cells or tissues, minimizing off-target effects and improving treatment efficacy. For example, they could be used to deliver gene therapy or RNA interference molecules to modulate disease-related pathways in PD and MS. These NPs can be functionalized with molecules that bind to specific biomarkers associated with neurological disorders. In addition, by detecting these biomarkers in biological fluids, such as blood or cerebrospinal fluid, NPs could enable early diagnosis and monitoring of disease progression.

One cohort study examined the benefits of using GNPs as a treatment for human subjects with PD and MS [3]. CNM-Au8 is a novel AuNC therapeutic agent that has been investigated for its potential to stimulate remyelination, particularly in neurological disorders such as MS and idiopathic PD. Preclinical studies involving mouse models of demyelination have shown that treatment with CNM-Au8 can promote remyelination. These studies typically include inducing demyelination in the central nervous system of mice and then administering CNM-Au8 to assess its effects on remyelination. Research suggests that CNM-Au8 treatment can enhance the recruitment and differentiation of OPCs responsible for remyelination. By promoting OPC proliferation and differentiation into mature oligodendrocytes, CNM-Au8 may facilitate the repair of damaged myelin sheaths [2]. In addition to promoting remyelination, CNM-Au8 treatment has been associated with improved functional outcomes in mouse models of demyelination. This includes improved motor function, coordination, and other neurological deficits associated with demyelinating diseases. The exact mechanisms underlying the remyelination-promoting effects of CNM-Au8 are not fully understood. However, it is believed that CNM-Au8 may exert its effects through various pathways, including modulation of cellular signaling pathways involved in OPC proliferation and differentiation and reducing oxidative stress and inflammation in the central nervous system.

Another study investigated the varying dosing needed for AuNCs to induce change in those diagnosed with PD. Mice were first induced with PD-like symptoms using MPTP and then treated with AuNCs at different doses (15). The mice were grouped into treatment groups, one receiving a low dose of AuNC (5 mg kg^-1^) and another receiving a high dose of AuNC (20 mg kg^-1^). Additionally, a control group that did not receive AuNC treatment was used to show symptom variations among the three groups. After AuNC treatment, the mice underwent behavioral tests to assess PD-like symptoms and motor function. These tests may include locomotor activity assessments, motor coordination tests (such as the rotarod test or beam-walking test), and other relevant assessments. Additionally, neurochemical analyses were conducted to evaluate the extent of DA neuron loss in the substantia nigra, and other brain regions, as well as changes in neurotransmitter levels. After injection with MPTP, the substantia nigra had reduced density in the mice; however, improvements in motor function, reductions in PD-like symptoms, and neuroprotection against DA neuron loss were seen when injecting the mice with a greater amount of gold NPs.

The potential use of gold NPs as a treatment for patients with PD and MS could have several significant implications for current and future patients. Gold NPs can be engineered to deliver therapeutic agents directly to injured areas of the brain or central nervous system, which could improve drug efficacy while reducing side effects [18]. This targeted drug delivery approach may lead to more effective treatments with fewer adverse reactions for patients with PD and MS. Gold NPs have shown promise in preclinical studies for their neuroprotective effects in PD and their potential to stimulate remyelination in MS. If these findings translate to clinical settings, gold NPs could help slow disease progression and preserve neurological function in patients. By targeting key pathological processes such as neuroinflammation, oxidative stress, and demyelination, gold NPs may have the potential to modify the underlying course of PD and MS. This could lead to treatments that alleviate symptoms and alter the natural history of these diseases, and thus offer long-term benefits for patients. Overall, the development of gold NP-based treatments for PD and MS represents a promising avenue for enhancing patients' healthcare in neurodegenerative and demyelinating diseases.

To further assess the safety and efficacy of gold NP-based treatments for PD and MS, several types of studies should be performed to investigate acute and chronic toxicity, biodistribution, and potential long-term effects in relevant animal models of PD and MS [19]. Particular attention should be paid to potential off-target effects, immunogenicity, and the risk of systemic toxicity. Pharmacokinetic studies are necessary to understand the absorption, distribution, metabolism, and excretion (ADME) of gold NPs in vivo. These studies can help determine optimal dosing regimens, identify potential accumulation in specific organs or tissues, and assess the potential for drug interactions or interference with endogenous biological processes. Long-term clinical trials are essential to evaluate the safety and efficacy of gold NP-based treatments over extended periods. By conducting these studies, researchers and clinicians can generate robust evidence to support the safe and effective use of gold NP-based treatments for PD and MS, ultimately improving patient care and outcomes in these debilitating neurological disorders.

Gold NPs have been investigated for biomedical applications beyond neurodegenerative disorders, including cancer diagnosis, imaging, therapy, and drug delivery [20]. They can be functionalized by targeting molecules that specifically bind to cancer cells or biomarkers, making them useful for cancer detection and diagnosis. Gold flakes can enhance the sensitivity and specificity of various diagnostic techniques, including imaging modalities like surface-enhanced Raman scattering (SERS) and photoacoustic imaging [21]. In addition, gold NPs have unique optical properties that make them excellent contrast agents for various imaging techniques. The gold flakes can improve the resolution and accuracy of tumor imaging, allowing for early detection, staging, and monitoring of cancer progression. Gadolinium is another elemental MRI contrast agent, which has been the subject of research due to evidence of its deposition in the globus pallidus and dentate nucleus after repeated and frequent use [22]. While this evidence is currently limited to the element gadolinium, further studies should likely be conducted to survey whether gold NPs would similarly deposit in these structures. Furthermore, gold NPs can also be used as vehicles for delivering therapeutic agents directly to cancer cells or tumors, minimizing systemic toxicity and improving treatment efficacy. Gold flakes can carry chemotherapy drugs, nucleic acids, or photothermal therapy agents to target tumors and inhibit their growth [23].

Citrate-coated gold NPs have specifically been shown to increase the targeting of trastuzumab antibodies to HER-2 receptors in human breast cancer cells [24]. Additionally, this study demonstrated that the gold NPs enhanced the drug-cell interaction of the trastuzumab antibodies once bound to the HER-2 receptors, thereby improving the drug’s efficacy and therapeutic effect. This finding could suggest gold NPs’ possible functionality as a synergistic therapy as opposed to simply a passive drug carrier. More research is needed to explore this claim.

Overall, gold flakes hold great promise for advancing not only the treatment of neurodegenerative disease but also cancer diagnosis, imaging, therapy, and drug delivery. Thus, gold flakes create innovative solutions to overcome challenges in cancer management and improve patient outcomes. Continued research and development are essential for translating these promising nanotechnologies into patient clinical applications (Table 1).

**Table 1:** Summary of clinical studies AD, Alzheimer’s disease; ALS, amyotrophic lateral sclerosis; AuNP, gold nanoparticle; GNP, gold nanoparticles; MS, multiple sclerosis; N/A, not applicable; NAD+, nicotinamide adenine dinucleotide; NADH, nicotinamide adenine dinucleotide + hydrogen; PD, Parkinson's disease; PO, by mouth; qAM, every day before noon; SN, substantia nigra

Author (year)	Disease	Type and duration of study	Number of patients in treatment group	Number of patients in control group	Dose/administration regimen of gold	Specific symptoms followed	Effect on symptoms (p-value if included)	Additional relevant data
Robinson et al. (2020) [2]	Animal models of MS	Experimental animal study (mice)	Unstated	Unstated	Unstated/variable amounts of CNM-Au8	Gross/fine motor movements	Improvement that typically did not reach statistical significance	Remyelination observed compared to the control group
Ren et al. (2023) [3]	PD	Cohort, 12 weeks	13	N/A	120 mL CNM-Au8 qAM PO, liquid	None	N/A	Increase in NAD+/NADH ratio by an average of 10.4% (p = 0.11)
Ren et al. (2023) [3]	MS	Cohort, 12 weeks	11	N/A	120 mL CNM-Au8 qAM PO, liquid	None	N/A	Increase in NAD+/NADH ratio by an average of 10.4% (p = 0.14)
Gao et al. (2019) [15]	Animal models of PD	Experimental animal study (mice)	Unstated	Unstated	Variable	Locomotor activities and motor coordination	Improvement in each measured category (p < 0.01)	AuNCs decreased α-Synuclein aggregation/fibrillation and reversed DA neuron loss in SN.
Hu et al. (2018) [16]	Animal models of PD	Experimental animal study (mice)	Unstated	Unstated	Intraabdominal GNP injections; variable/unstated amounts	Gait test (mentioned in passing; the focus of the study was effects at the molecular level and on brain imaging)	Improvement; further details/statistical significance (or lack thereof) unstated	Multiple improvements observed at the molecular level and on brain imaging
Tramontin et al. (2020) [17]	Animal models of AD	Experimental study (rats)	12	12	20-nm AuNP (at a dose of 2.5 mg/kg) every 48 hours for 21 days	Spatial memory	Improvement; further details/statistical significance unstated in abstract	Multiple improvements observed at the molecular level and on brain imaging
Vucic et al. (2023)	ALS	Phase 2, randomized, double-blind, placebo-controlled trial; 36 weeks	23	22	30 mg CNM-Au8 daily, orally or by feeding tube; liquid	Mean percent changed in summed motor unit number index (MUNIX, biomarker of LMN function)	Slower decline in summated MUNIX score compared to control group (p = 0.0783)	60% reduction in all-cause mortality at 12 months with CNM-Au8 treatment; slower rate of disease progression observed

Related to the continued rise in worldwide life expectancy and more advanced diagnostic technology, the prevalence of neurodegenerative disorders continues to rise. It is expected to grow in the coming years [25-27]. However, despite the growing burden of neurodegenerative disorders, prognoses remain poor, especially for Alzheimer’s and ALS. Treatment options to reverse damage and restore function are limited or unavailable and restricted mainly to supportive or palliative care [28-30]. As of 2023, there has been newfound intrigue in the promising use of monoclonal antibody treatments against Aβ plaques in the management of AD progression; however, the use of this therapy in human patients has so far been limited by a lack of sufficient animal models [31].

## Conclusions

The treatments that are currently available carry undesirable and uncomfortable adverse effects. For these reasons, the treatment of neurological disorders with GNPs is a necessary field of study, yielding promising results in lab, animal, and human studies. GNPs, specifically CNM-Au8, have been shown to increase NADH conversion to NAD+ in PD, protect against Aβ accumulation in AD, promote remyelination in MS, and decrease all-cause mortality in ALS. These experimental results hold significance for patients with neurodegenerative disorders. Gold particles have the potential to slow disease progression and reverse neuronal cellular damage. NPs were not associated with any major adverse effects during trials, meaning this therapy is potentially both effective and safe. Large-scale human clinical trials need to be conducted to examine safety and efficacy further. Using GNPs, such as CNM-Au8, potentially improves motor function and memory, prevents further neurologic damage, reverses damage, and raises life expectancy for those with neurological disorders.
